# Histone Deacetylase Inhibitors Inhibit the Proliferation of Gallbladder Carcinoma Cells by Suppressing AKT/mTOR Signaling

**DOI:** 10.1371/journal.pone.0136193

**Published:** 2015-08-19

**Authors:** Peng Zhang, Zhiyong Guo, Ying Wu, Ronglin Hu, Jun Du, Xiaoshun He, Xingyuan Jiao, Xiaofeng Zhu

**Affiliations:** 1 Organ Transplant Center, The First Affiliated Hospital, Sun Yat-sen University, Guangzhou, China; 2 Department of Biostatistics, The First Affiliated Hospital, Sun Yat-sen University, Guangzhou, China; 3 Department of Microbial and Biochemical Pharmacy, School of Pharmaceutical Sciences, Sun Yat-sen University, Guangzhou, China; Peking University Health Science Center, CHINA

## Abstract

Gallbladder carcinoma is an aggressive malignancy with high mortality mainly due to the limited potential for curative resection and its resistance to chemotherapeutic agents. Here, we show that the histone deacetylase inhibitors (HDACIs) trichostatin-A (TSA) and suberoylanilide hydroxamic acid (SAHA) reduce the proliferation and induce apoptosis of gallbladder carcinoma cells by suppressing the AKT/mammalian target of rapamycin (mTOR) signaling. Gallbladder carcinoma SGC-996 cells were treated with different concentrations of TSA and SAHA for different lengths of time. Cell proliferation and morphology were assessed with MTT assay and microscopy, respectively. Cell cycle distribution and cell apoptosis were analyzed with flow cytometry. Western blotting was used to detect the proteins related to apoptosis, cell cycle, and the AKT/mTOR signaling pathway. Our data showed that TSA and SAHA reduced SGC-996 cell viability and arrested cell cycle at the G1 phase in a dose- and time-dependent manner. TSA and SAHA promoted apoptosis of SGC-996 cells, down-regulated the expression of cyclin D1, c-Myc and Bmi1, and decreased the phosphorylation of AKT, mTOR p70S6K1, S6 and 4E-BP1. Additionally, the mTOR inhibitor rapamycin further reduced the cell viability of TSA- and SAHA-treated SGC-996 cells and the phosphorylation of mTOR, whereas the mTOR activator 1,2-dioctanoyl-sn-glycero-3-phosphate (C8-PA) exerted the opposite influence. Our results demonstrate that histone deacetylase inhibitors (HDACIs) suppress the proliferation of gallbladder carcinoma cell via inhibition of AKT/mTOR signaling. These findings offer a mechanistic rationale for the application of HDACIs in gallbladder carcinoma treatment.

## Introduction

Gallbladder carcinoma is the fifth most commonly diagnosed gastrointestinal malignancy worldwide and the most aggressive malignant neoplasm of the biliary tract [1–2]. Mainly due to its non-specific symptoms and highly invasive nature, most patients are diagnosed at an advanced stage, with only 20%-40% of patients suitable for curative resection [3]. The prognosis of gallbladder carcinoma is notoriously poor. The median survival period of gallbladder carcinoma patients is less than one year, while the 5-year survival rate is approximately 5% [4, 5]. In addition, the efficacy of current adjuvant chemotherapy and radiotherapy of gallbladder cancer is minimal [6]. Therefore, it is an urgent task to elucidate the precise molecular mechanism of gallbladder carcinoma development and identify novel and effective targets for the development of anticancer agents for the treatment of gallbladder carcinoma.

Histone deacetylases (HDACs) are a group of enzymes that remove acetyl groups from histones and alter chromatin metabolisms such as DNA replication and gene transcription. HDACs play a crucial role in the regulation of cell proliferation and cell death. Aberrant patterns of histone acetylation maintain the transformed state of human tumor cells, which can be reversed by inhibiting HDACs. There is a growing body of evidence showing that HDACs are up-regulated in a variety of cancers [7]. This makes HDAC inhibitors (HDACIs) promising potential targeted anticancer agents and numerous HDACIs are currently in preclinical and clinical trials. Moreover, normal cells are relatively more resistant to HDACI-induced cell death than cancer cells [8]. Indeed, vorinostat (suberoylanilide hydroxamic acid; SAHA) and trichostatin-A (TSA) have shown strong anti-proliferative effects and protective ability against intracellular events in different cells and cancers [9–12]. SAHA inhibits all the class I and II HDAC family members, and leads to specific modifications of acetylation and methylation of lysines [13]. SAHA is currently one of the most advanced agents in clinical development of cancer therapeutics due to its low toxicity, and was approved by the U.S. Food and Drug Administration for the treatment of cutaneous T-cell lymphoma [14]. However, the effects of HDACIs on gallbladder carcinoma cells and the underlying mechanisms are not well understood.

To explore the potential of HDACIs for the treatment of gallbladder carcinoma, we have assessed the effects of TSA and SAHA on the growth and proliferation of gallbladder carcinoma SGC-996 cells. We found that TSA and SAHA suppressed the proliferation of SGC-996 cells and arrested cell cycle at the G1 phase, accompanied with suppression of the AKT/mammalian target of rapamycin (mTOR) signaling.

## Materials and Methods

### Chemicals and reagents

The histone deacetylase inhibitors TSA and SAHA, and the mammalian target of rapamycin complex 1 (mTORC1) inhibitor rapamycin were purchased from Sigma-Aldrich (St. Louis, MO, USA) and dissolved in dimethyl sulfoxide (DMSO; Sigma-Aldrich). 1,2-dioctanoyl-sn-glycero-3-phosphate (C8-PA) was purchased from Avanti Lipids (Alabaster, AL, USA) and dissolved in DMSO. Primary antibodies against AKT (pan), phospho-AKT (Ser473), mTOR, phospho-mTOR (Ser2448), p70 S6 kinase, phospho-p70 S6 kinase (Thr389), S6 ribosomal protein, phospho-S6 ribosomal protein (Ser235/236), 4E-BP1, phospho-4E-BP1 (Thr37/46), acetyl-histone H3 (Lys9), Bmi1, cyclin D1, and c-Myc were obtained from Cell Signaling Technology (Beverly, MA, USA). Primary antibodies against Bax, Bcl-2 and β-actin were obtained from Santa Cruz Biotechnology (Santa Cruz, CA, USA). Horseradish peroxidase (HRP)-conjugated secondary antibodies (goat-anti-rabbit and goat-anti-mouse) were purchased from Invitrogen (Carlsbad, CA, USA).

### Cell culture

The human primary gallbladder carcinoma cell line SGC-996 was purchased from the Academy of Life Science, Tongji University (Shanghai, China) [15] and maintained in RPMI-1640 medium (Gibco BRL) supplemented with 10% fetal bovine serum and 1% penicillin-streptomycin at 37°C in a 5% CO_2_ incubator.

### Cell viability assay and cell density analysis

To assess the changes in cell viability after drug treatment, a 3-(4,5-dimethylthiazol-2-yl)-2,5-diphenyl tetrazolium bromide (MTT; Sigma-Aldrich) assay was performed. In brief, exponentially growing cells were plated in triplicate at 4×10^3^ cells per well into 96-well plates and grown overnight. The cells were subjected to the following drug treatments: rapamycin (10, 50, 200, 500 and 1000 nM) for 24, 48, or 72 h; TSA (0.025, 0.05, 0.1, 0.2, 0.4, 0.8, 1.6, and 3.2 μM) or SAHA (0.312, 0.625 1.25, 2.5, 5, 10, 20, and 40 μM) for 48 h, or 0.4 μM TSA or 10 μM SAHA for the indicated time. At the end of experiments, 20 μl of 5 mg/ml MTT was added to each well and the cells were incubated at 37°C for an additional 4 h. The supernatant was then discarded and the resulting formazan crystals were dissolved by adding 150 μl DMSO to each well and incubated for 10 min at 37°C. The optical density was measured using a Vmax Microplate Reader (Molecular Devices, Silicon Valley, CA, USA) at 490 nm. The percent cell viability was calculated using the following formula: percent cell viability = [(absorbance of the experimental well)—(absorbance of the blank)] / [(absorbance of untreated control well)—(absorbance of blank)] ×100%. The 50% inhibitory concentration (IC_50_) was calculated by a Logit model based on the cell viability data. To measure cell density after TSA or SAHA treatment, the cell number was counted in each group. The average cell number was obtained from three independent experiments. The experiments were performed in triplicate and repeated at least twice.

### Flow cytometry

SGC-996 cells were plated in 6-well plates at a density of 3×10^5^ cells per well and incubated for 24 h at 37°C. For cell cycle analysis, sub-confluent cells were treated with or without various concentrations of TSA (0.1 and 0.4 μM) or SAHA (1, 2.5 and 5 μM) for 48 h. The cells were then harvested, washed twice with ice-cold phosphate-buffered saline (PBS) for cell cycle and apoptosis detection. For cell cycle distribution analysis, cells were fixed with 70% ethanol, treated with 1% RNase, and stained with propidium iodide (100 μg/ml final concentration; Sigma-Aldrich). Cell apoptosis was assayed using Annexin V Apoptosis Detection Kit I (BD Biosciences) according to the manufacturer’s instructions. Cells at a density of 1×10^6^ cell/ml were resuspended with 1× binding buffer and then 100 μl of the resulting cell suspension was mixed with 5 μl Annexin V and 5 μl 7-AAD. The samples were then analyzed for the proportion of apoptotic cells using a FACS scanner (Becton Dickinson) and the software FlowJo (Tree Star, Ashland, OR, USA). Sub-G1 cells identified in flow cytometric histograms were considered apoptotic cells.

### Protein extraction and Western blotting analysis

Protein extraction and Western blotting were performed as we described previously [16]. Briefly, cells were washed three times with ice-cold phosphate buffer and lysed in a lysis buffer. 30 μg of total protein were separated on 10% SDS–polyacrylamide gels and then transferred onto PVDF membranes. Subsequently, the membranes were blocked with 5% non-fat milk at room temperature for 2 h and incubated with primary antibodies at 4°C overnight, followed by incubation with a HRP-conjugated secondary antibody for 1 h at room temperature. Signals were detected with the Western Blotting Plus Chemiluminescence Reagent (Life Science, Inc., Boston, MA).

### Statistical analysis

The data are represented as mean ± standard deviation (SD) and are from three independent experiments unless otherwise specified. Data were analyzed by two-tailed unpaired Student’s t-test between any two groups. Statistical analyses were performed using SPSS statistics version 13.0 (SPSS Inc., USA) by ANOVA followed by the Student-Newman-Keuls (S-N-K) method for the multiple comparisons. Differences were considered statistically significant at *P*< 0.05.

## Results

### HDACIs inhibited the proliferation of gallbladder carcinomaSGC-996 cells in a dose- and time-dependent manner

**Fig 1:**
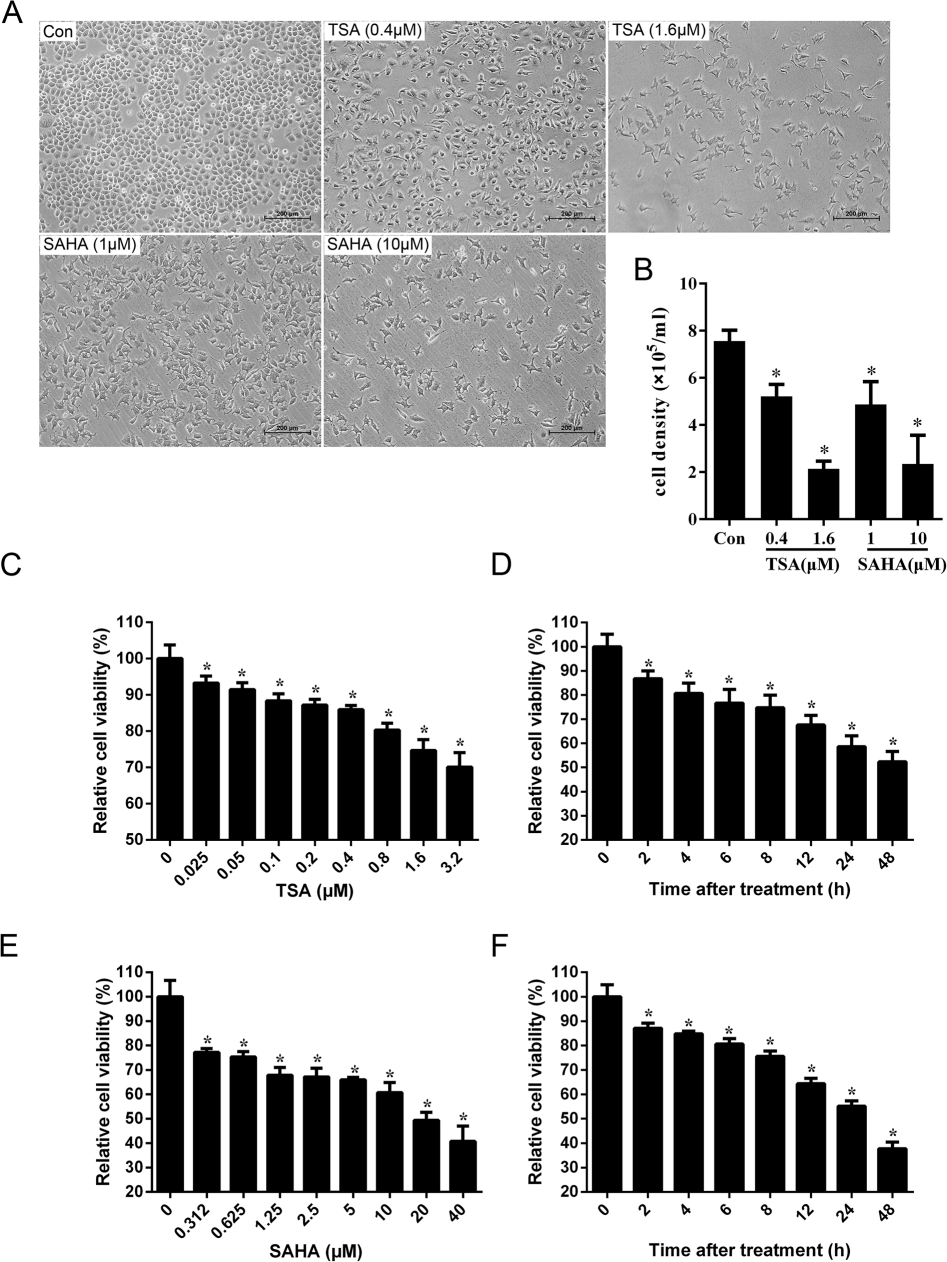
HDACIs reduced the cell viability of gallbladder carcinoma cells in a dose- and time-dependent manner. SGC-996 cells were treated with or without different concentrations of TSA or SAHA for 24 h. The cell morphology (A) and cell number (B) were recorded under a phase contrast microscope. Scale bars: 200 μm. (C) SGC-996 cells were treated with various concentrations of TSA for 48 h. Cell viability was measured by MTT assay. (D) SGC-996 cells were incubated with 0.4 μM TSA for the time indicated. Cell viability was measured by MTT assay. (E) SGC-996 cells were treated with various concentrations of SAHA for 48 h. Cell viability was measured by MTT assay. (F) SGC-996 cells were incubated with 10 μM SAHA for the time indicated. Cell viability was measured by MTT assay. The data are representatives of three independent experiments with SD for triplicates. Compared with the control group at 0 μM (h): *P< 0.05.

In this study, gallbladder carcinoma SGC-996 cells were initially treated with the HDACIs TSA (0.4 and 1.6 μM) or SAHA (1 and 10 μM) for 24 h, and then the number of cells was recorded and morphological changes observed by phase contrast microscopy. Under normal growth conditions, SGC-996 cells grew with adherence and were tightly bound as a homogeneous polygon shape, with the typical morphological characteristics of epithelial cells. In contrast, HDACI-treated cells were partly spindle-shaped with extended pseudopodia, an indication of apoptosis (Fig 1A). Compared to the untreated control, the number of SGC-996 cells decreased with increasing concentrations of either TSA or SAHA (Fig 1A and 1B). These results indicate that HDACI treatment of SGC-996 cells leads to loss of cell viability.

To further support this observation, cell proliferation was assessed by MTT assay and SGC-996 cells were found to be sensitive to HDACI treatment, especially SAHA. Specifically, TSA at 0.025 μM and SAHA at 0.312 μM obviously reduced cell viability (Fig 1C and 1E). Treatment with 0.4 μM TSA and 10 μM SAHA for 2 h reduced cell viability to 87.1% and 86.8%, respectively. We also found that the inhibitory effects of SGC-996 cell proliferation increased as the duration of drug exposure lengthened, especially after SAHA treatment (Fig 1D and 1F). The IC_50_ of TSA and SAHA was 38.14 and 22.13 μM, respectively. Thus, HDACIs reduced the cell viability of gallbladder carcinoma cells in a dose- and time-dependent manner.

### HDACIs induced the arrest of SGC-996 cells in the G1 phase of the cell cycle in a dose-dependent manner

**Fig 2:**
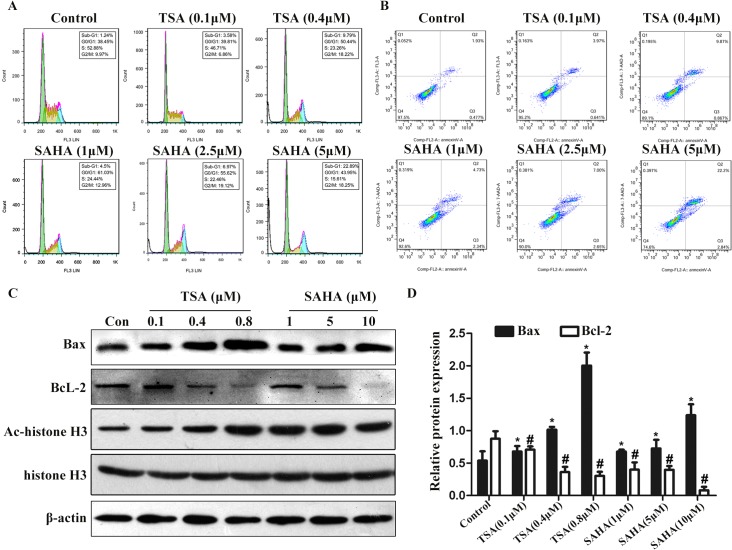
HDACIs induced the arrest in the G1 phase of the cell cycle and apoptosis of SGC-996 cells. SGC-996 cells were treated with or without TSA (0.1 or 0.4 μM) or SAHA (1, 2.5 or 5 μM) for 48 h and then subjected to flow cytometric assay for cell cycle analysis (A) and apoptosis detection (B). (C) SGC-996 cells were incubated with or without TSA or SAHA at the indicated concentration for 24 h. Total proteins were extracted for immunoblotting of Bcl-2, Bax, Ac-histone 3 and total histone 3 with β-actin as the loading control. (D) Analysis of band intensity of Bcl-2 and Bax on C. Data shown represent three independent experiments *P< 0.05 vs. the expression of Bax in the control group. #*P*< 0.05 vs. the expression of Bcl-2 in the control group.

To determine the underlying mechanism by which HDACIs inhibit SGC-996 cell proliferation, cell cycle distribution was analyzed. Compared to untreated controls, TSA (0.1 and 0.4 μM) and SAHA (1, 2.5 and 5 μM) treatment for 48 h resulted in apparent accumulation of SGC-996 cells at the G1 phase of the cell cycle in a dose-dependent manner. Sub-G1 cells were considered apoptotic cells. Either 0.1 μM TSA or 1 μM SAHA treatment for 48 h significantly induced accumulation of SGC-996 cells in the sub-G1 phase of the cell cycle by 3.58% and4.5% respectively, compared to the untreated control (1.24%). Furthermore, with increasing drug concentrations, the number of sub-G1 cells accumulated significantly (Fig 2A). Moreover, cells treated with TSA (0.1 and 0.4 μM) or SAHA (1, 2.5 and 5 μM) displayed a higher proportion of apoptotic cells than the untreated control (Fig 2B). Similar to the results of the cell cycle assay, as the concentrations of TSA or SAHA increased, the percentage of apoptotic cells also increased. As expected, Western blot analyses showed that either TSA or SAHA obviously increased the level of Acyt-histone 3 (Fig 2C). However, the level of anti-apoptotic Bcl-2 protein was measurably lower after 0.4 μM TSA or 5 μM SAHA treatments for 24 h, whereas the level of pro-apoptotic Bax protein was up-regulated (Fig 2C and 2D). Moreover, the changes in both Bcl-2 and Bax protein levels were more significant when the concentrations of TSA and SAHA were increased. Taken together, these results clearly demonstrated that HDACIs induced G1-phase cell cycle arrest and apoptosis in a dose-dependent manner in gallbladder carcinoma cells.

### HDACIs down-regulated protein levels of cyclin D1, c-Myc and Bmi1 and up-regulated histone H3 acetylation

**Fig 3:**
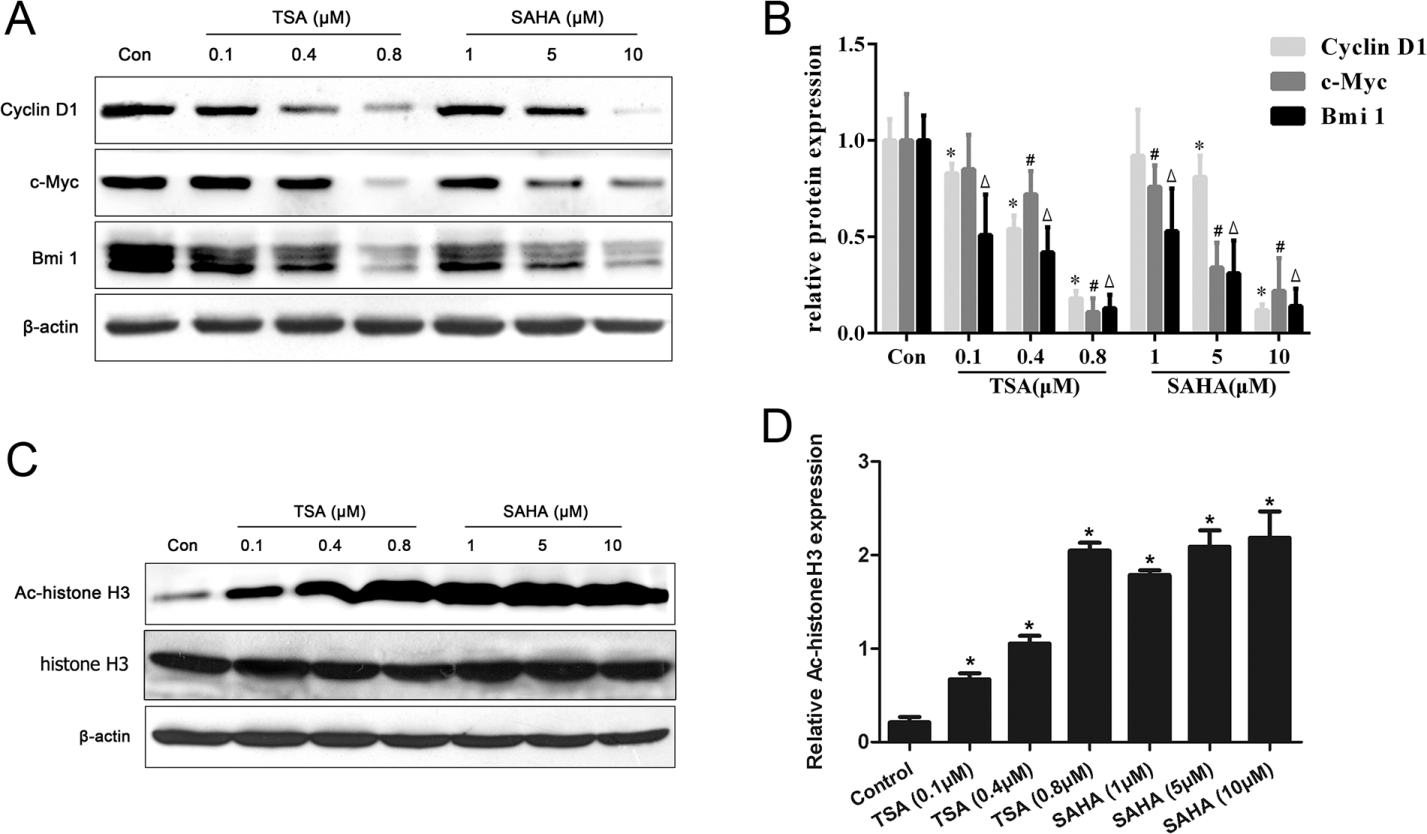
HDACIs decreased cyclin D1, c-Myc and Bmi1 and increased histone H3 acetylation. (A) SGC-996 cells were treated with different concentrations of TSA or SAHA as indicated for 24 h. Total proteins were extracted for immunoblotting of cyclin D1, c-Myc and Bmi1with β-actin as the loading control. (B) Analysis of band intensity in A. *P< 0.05 vs. cyclin D1 in control. #*P*< 0.05 vs. c-Myc in control. Δ*p*< 0.05 vs Bmi1 in control. (C) Cells were treated as in A; histone H3 acetylation was determined by Western blots with β-actin as the loading control. (D) Analysis of band intensity in C. *P< 0.05 vs. control. Data shown represent three independent experiments.

To look into the underlying molecular mechanism of the anti-proliferative and pro-apoptotic activities of HDACIs in SGC-996 cells, we determined the expression of cyclin D1, c-Myc and Bmi1, which play critical roles in regulating cell proliferation and apoptosis. It was found that TSA and SAHA dose-dependently down-regulated the protein levels of cyclin D1, c-Myc and Bmi1 (Fig 3A and 3B). Acetyl-histone H3 is a well-known target of SAHA [17]. Indeed, both TSA and SAHA up-regulated the levels of acetyl-histone H3 protein in a dose-dependent manner (Fig 3C and 3D). These results indicate that TSA and SAHA might prevent G1-to-S phase transition by decreasing the expression of cyclin D1, c-Myc, and Bmi1 in SGC-996 cells.

### HDACIs suppressed the activity of AKT/mTOR signaling and its downstream targets

**Fig 4:**
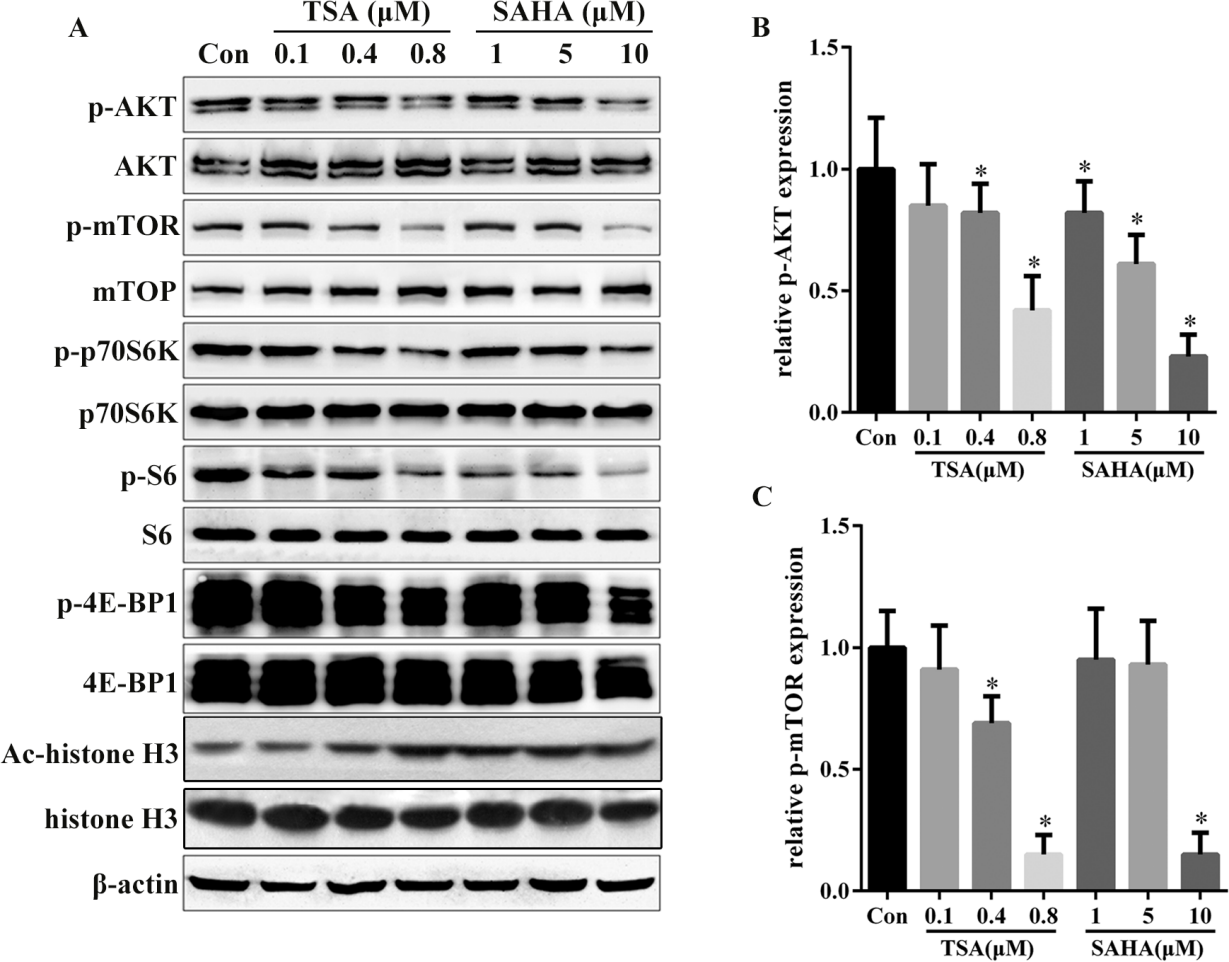
HDACIs inhibited the activity of AKT/mTOR signaling. (A) SGC-996 cells were treated with 0.1, 0.4 and 0.8 μM TSA or 1, 5 and 10 μM SAHA for 24 h. Total proteins were extracted for immunoblotting with antibodies against the indicated proteins. β-actin served as the loading control.(B) Relative band intensity of p-AKT in A. (C) Relative band intensity of p-mTOR in A. Data shown represent three independent experiments. *P< 0.05 vs. control.

The AKT/mTOR signaling pathway is a prominent cell-growth promoting pathway that is deregulated in most cancers. Pharmacological inhibition of AKT/mTOR signaling results in cell cycle arrest at the G1 phase and induction of cell apoptosis. Cyclin D1, c-Myc, and Bmi1 are the downstream targets of AKT/mTOR signaling. Our observation that treatment with TSA and SAHA leads to cell cycle arrest at the G1 phase as well as induction of apoptosis, accompanied by a decrease in the levels of cyclin D1, c-Myc, and Bmi1, suggests that inhibition of HDACs by TSA and SAHA may suppress the activity of the AKT/mTOR signaling pathway. Indeed, after 24 h of treatment, 0.8 μM TSA and 10 μM SAHA dramatically diminished the levels of phosphorylated AKT protein without modulation of the total amount of AKT (Fig 4A and 4B). Similarly, treatment of SGC-996 cells with 0.8 μM TSA or 10 μM SAHA for 24 h effectively down-regulated levels of the phosphorylated form of mTOR (Fig 4A and 4C). In addition, the phosphorylation of p70S6K, S6 and 4E-BP1, all of which are markers of the activity of mTOR signaling, was clearly and dose-dependently suppressed by both TSA and SAHA, accompanied with upregulation of the acetylation of histone 3 (Fig 4A).

### Rapamycin and C8-PA affects the phosphorylation of mTOR and the viability of HDACIs-treated gallbladder carcinoma cells

**Fig 5:**
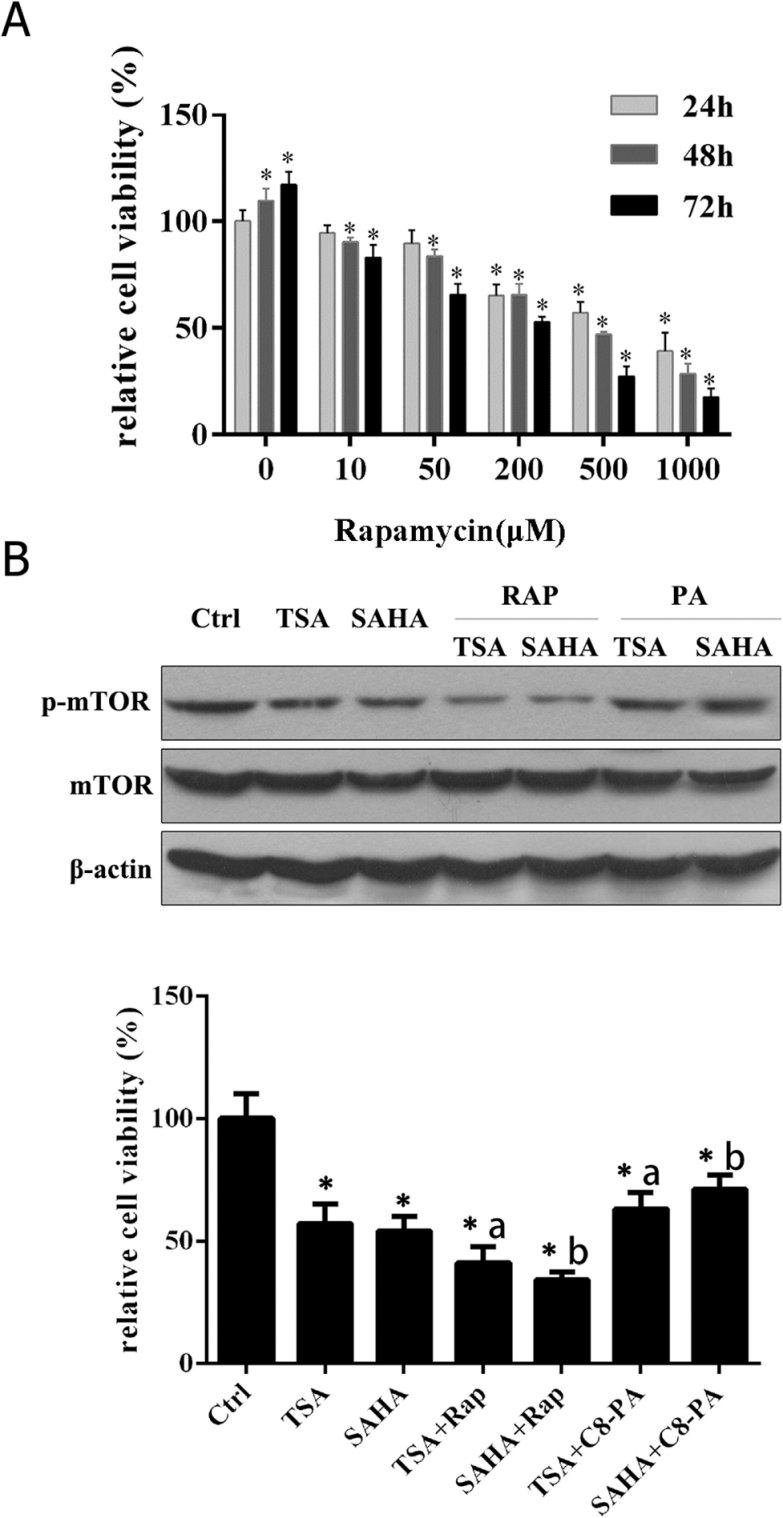
mTOR signaling involved in HDACIs-induced cell proliferation inhibition. (A) SGC-996 cells were treated with the indicated concentrations of rapamycin for 24, 48 or 72 h. Cell viability was measured using an MTT assay. *P< 0.05 vs. 24 h of the 0 nM group. (B) Western blot showing that p-mTOR expression and cell viability were affected by rapamycin and C8-PA in the HDACIs treated SGC-996 cells. Ctl, untreated control cells; TSA, cells treated with 0.4μM TSA; SAHA, cells treated with 10μM SAHA. RAP, rapamycin (50μM); PA, C8-PA (300μM). *P< 0.05 vs. Ctrl; a, *P*< 0.05 vs. TSA group; b, *P*< 0.05 vs. SAHA group.

mTOR kinase is the central integrator and regulator of multiple intracellular signal pathways. Numerous inhibitors of mTOR signaling pathways are undergoing preclinical and clinical trials for the treatment of a wide range of cancers. Among these inhibitors, rapamycin is a well-known agent. To test whether rapamycin’s inhibition of mTOR signaling leads to a decrease in cell growth and in the proliferation of gallbladder carcinoma cells, SGC-996 cells were treated with different concentrations of rapamycin for 24, 48, and 72 h, with cell viability subsequently determined by MTT assay. Our results showed that rapamycin significantly reduced SGC-996 cell viability in a dose- and time-dependent manner (Fig 5A). The IC_50_ of rapamycin in SGC-996 cells was 854.1 μM for 24 h, 381.4 μM for 48 h, and 156.4 μM for 72 h. Thus, rapamycin is a promising agent in the treatment of gallbladder carcinoma.

In order to assess whether the observed apoptotic effect of HDACIs is related to mTOR pathway signaling, SGC-996 cells were treated with rapamycin or C8-PA to deactivate or activate p-mTOR, respectively. Western blot results showed that cells treated with TSA or SAHA down-regulated p-mTOR expression (Fig 5B). Moreover, treatment with rapamycin (50 μM) + TSA (0.4 μM), or rapamycin (50 μM) + SAHA (10 μM) treatment, further suppressed the expression level of p-mTOR, which was reversed by C8-PA (300 μM) in both TSA (0.4 μM)- and SAHA (10 μM)-treated groups. Consistent with p-mTOR expression, MTT assays showed that rapamycin decreased the cell viability of TSA- and SAHA-treated groups, whereas C8-PA was able to partly reverse the inhibitory effect of TSA or SAHA treatment (Fig 5B).

## Discussion

In this study, we showed that treatment of gallbladder carcinoma SGC-996 cells with the HDAC inhibitors TSA and SAHA resulted in loss of cell viability and induction of apoptosis, accompanied with G1-phase cell cycle arrest. Moreover, TSA and SAHA down-regulated the protein levels of cyclin D1, c-Myc and Bmi1, and suppressed the activity of AKT/mTOR signaling. Meanwhile, the mTOR inhibitor rapamycin reduced SGC-996 cells’ viability in a dose- and time-dependent manner. Our findings suggest that HDAC inhibitors TSA and SAHA are promising agents for the treatment of gallbladder carcinoma.

Gallbladder carcinoma is a lethal disease with only a minority of patients suitable for tumor resection. To date, no successful alternative therapy has been developed, mainly because gallbladder cancer cells are relatively less sensitive to conventional chemotherapy and radiotherapy [18]. Hence, new, effective treatments and safe drugs are urgently needed to improve the outcome of patients with advanced gallbladder carcinoma. Because uncontrolled proliferation and evasion of apoptosis are two hallmarks of cancer cells, inhibition of proliferation and induction of apoptosis by small molecules may be a novel strategy for the treatment of gallbladder carcinoma. Recent studies have demonstrated that HDACIs induce growth arrest, activate extrinsic and/or intrinsic apoptotic pathways, and cause autophagic cell death, mitotic cell death and senescence in several types of cancer cells [19]. In the current study, it was found, for the first time, that HDACIs inhibited the proliferation and induced apoptosis of gallbladder carcinoma cells *in vitro* by suppressing AKT/mTOR signaling.

In recent years, the anti-cancer activity of HDACIs has been widely investigated in diverse cancer cells, but their effects on gallbladder carcinoma cells have remained largely unknown. It has been reported that SAHA, in combination with the poly (ADP-ribose) polymerase (PARP) inhibitor olaparib, demonstrated a synergistic reduction of prostate cancer cell viability and induction of apoptosis compared to single agent treatment, while normal prostatic cells were not affected [20]. Yamaguchi et al. [21] found that SAHA treatment caused a significant inhibition of cell proliferation in gallbladder carcinoma TGBC2TKB cells and cholangiocarcinoma TFK-1 and HuCCT-1 cells, whereas normal cells were not sensitive to SAHA. Bajbouj et al. [22] showed that TSA slightly inhibited the proliferation and viability of glioblastoma U87 cells with accumulation of cells in the G1/S phase, but without apparent apoptosis. This different effect on apoptosis may result from different cell lines used. Another recent study showed that the histone deacetylase inhibitor PCI-24781 potently inhibited the growth and induced apoptosis of biliary tract cancer cells by decreasing the expression and activity of erbB2 [23]. Consistent with these previous studies, we found the cell viability of gallbladder carcinoma SGC-996 cells was significantly reduced after treatment with TSA and SAHA, accompanied with cell cycle arrest at the G1 phase and induction of apoptosis. Moreover, the level of the anti-apoptotic Bcl-2 protein was reduced, whereas the level of the pro-apoptotic Bax protein was up-regulated after treatment with TSA and SAHA. These data together indicate that HDACIs inhibit cell proliferation by G1-phase cell cycle arrest and induce apoptosis in gallbladder carcinoma cells.

Furthermore, the current study revealed that HDACIs are able to inhibit AKT/mTOR signaling and its downstream targets, and that the mTOR inhibitor rapamycin reduces the viability of gallbladder carcinoma cells. mTOR is a serine/threonine protein kinase that plays a central role in the regulation of cell growth, differentiation and apoptosis [24]. mTOR forms two distinct complexes, mTORC1 and mTORC2 [25]. mTORC1 is a downstream target of the phosphatidylinositol 3 kinase (PI3K)/AKT signaling pathway, which promotes cell survival and proliferation [26]. AKT directly phosphorylates mTOR to activate mTORC1, which in turn phosphorylates several downstream targets including the best-characterized 4E-BP1 and p70S6K. Phosphorylation of 4E-BP1 by mTORC1 results in release of eIF4E, allowing the initiation of cap-dependent protein translation. At the same time, mTORC1 facilitates ribosome biogenesis and translation elongation by phosphorylating p70S6K1. The phosphorylation status of p70S6K is commonly used as a marker of mTORC1 activity. Activation of mTOR increases translation of mRNAs with long and highly structured 5'-untranslated regions, such as cyclin D1 and c-Myc [27, 28]. In agreement with our findings, Nishiokaet al. [29] showed that the HDAC inhibitor MS-275 was able to block Akt/mTOR signaling in acute myelogenous leukemia HL60 cells and acute promyelocytic leukemia cells. Huang et al. [30] reported that another HDAC inhibitor, NBM-HD-3, exhibits anti-cancer activity through modulation of PTEN and AKT in brain cancer cells. Several other studies have also reported that HDACIs can inhibit the PI3K/AKT/mTOR signaling pathway in cancer cells [17, 31–33]. In the current study, rapamycin further decreased the phosphorylation of mTOR in the HDACI-treated cells, while the suppressive effect of HDACIs was reversed by C8-PA, an mTOR activator. Thus, inhibition of the PI3K/AKT/mTOR signaling axis is one of the mechanisms by which HDACIs reduce the proliferation and induce apoptosis of cancer cells.

Taken together, we have for the first time demonstrated that HDACIs inhibit the viability of gallbladder carcinoma cells in a dose- and time-dependent manner and cause G1 phase arrest of the cell cycle by suppressing AKT/mTOR signaling. Our findings provide a mechanistic rationale for the development of HDACIs as a single therapy, or in combination with mTOR inhibitors, in the treatment of gallbladder carcinoma.

## References

[1] Coburn NG, Cleary SP, Tan JC, Law CH. Surgery for gallbladder cancer: a population-based analysis. J Am Coll Surg 2008; 207: 371–382. 10.1016/j.jamcollsurg.2008.02.031 18722943

[2] Zhang P, Jiang G, Gao J, Li L, Du J, Jiao X. SAHA down-regulates the expression of indoleamine 2,3-dioxygenase via inhibition of the JAK/STAT1 signaling pathway in gallbladder carcinoma cells. Oncol Rep 2013; 29: 269–275. 10.3892/or.2012.2073 23042548

[3] Batra Y, Pal S, Dutta U, Desai P, Garg PK, Makharia G, et al Gallbladder cancer in India: a dismal picture. J Gastroenterol Hepatol 2005; 20: 309–314. 15683437 10.1111/j.1440-1746.2005.03576.x

[4] Bartlett DL, Fong Y, Fortner JG, Brennan MF, Blumgart LH. Long-term results after resection for gallbladder cancer. Implications for staging and management. Ann Surg 1996; 224: 639–646. 8916879 10.1097/00000658-199611000-00008 PMC1235441

[5] Butte JM, Matsuo K, Gönen M, D'Angelica MI, Waugh E, Allen PJ, et al Gallbladder cancer: differences in presentation, surgical treatment, and survival in patients treated at centers in three countries. J Am Coll Surg 2011; 212: 50–61. 10.1016/j.jamcollsurg.2010.09.009 21075015

[6] Czito BG, Hurwitz HI, Clough RW, Tyler DS, Morse MA, Clary BM, et al Adjuvant external-beam radiotherapy with concurrent chemotherapy after resection of primary gallbladder carcinoma: a 23-year experience. Int J Radiat Oncol Biol Phys 2005; 62: 1030–1034. 15990005 10.1016/j.ijrobp.2004.12.059

[7] Witt O, Deubzer HE, Milde T, Oehme I. HDAC family: What are the cancer relevant targets? Cancer Lett 2009; 277: 8–21. 10.1016/j.canlet.2008.08.016 18824292

[8] Burgess A, Ruefli A, Beamish H, Warrener R, Saunders N, Johnstone R, et al Histone deacetylase inhibitors specifically kill nonproliferating tumour cells. Oncogene 2004; 23: 6693–6701. 15235588 10.1038/sj.onc.1207893

[9] Batty N, Malouf GG, Issa JP. Histone deacetylase inhibitors as anti-neoplastic agents. Cancer Lett 2009; 280: 192–200. 10.1016/j.canlet.2009.03.013 19345475

[10] Hahnen E, Hauke J, Tränkle C, Eyüpoglu IY, Wirth B, Blümcke I. Histone deacetylase inhibitors: possible implications for neurodegenerative disorders. Expert Opin Investig Drugs 2008; 17: 169–184. 10.1517/13543784.17.2.169 18230051

[11] Mwakwari SC, Patil V, Guerrant W, Oyelere AK. Macrocyclic histone deacetylase inhibitors. Curr Top Med Chem 2010; 10: 1423–1440. 20536416 10.2174/156802610792232079 PMC3144151

[12] Monneret C. Histone deacetylase inhibitors for epigenetic therapy of cancer. Anticancer Drugs 2007; 18: 363–370. 17351388 10.1097/CAD.0b013e328012a5db

[13] Bhalla KN. Epigenetic and chromatin modifiers as targeted therapy of hematologic malignancies. J Clin Oncol 2005; 23: 3971–3993. 15897549 10.1200/JCO.2005.16.600

[14] Duvic M, Vu J. Update on the treatment of cutaneous T-cell lymphoma (CTCL): Focus on vorinostat. Biologics 2007; 1: 377–392. 19707308 PMC2721288

[15] Lin W, Jiang L, Chen Y, Li L, Du J, Jiao X. Vascular endothelial growth factor-D promotes growth, lymphangiogenesis and lymphatic metastasis in gallbladder cancer. Cancer Lett 2012; 314: 127–136. 10.1016/j.canlet.2011.09.004 22071224

[16] Zhang P, Jiang G, Gao J, Li L, Du J, Jiao X. SAHA down-regulates the expression of indoleamine 2, 3-dioxygenase via inhibition of the JAK/STAT1 signaling pathway in gallbladder carcinoma cells. Oncol Rep 2013; 29: 269–275. 10.3892/or.2012.2073 23042548

[17] Kawamata N, Chen J, Koeffler HP. Suberoylanilide hydroxamic acid (SAHA; vorinostat) suppresses translation of cyclin D1 in mantle cell lymphoma cells. Blood 2007; 110: 2667–2673. 17606765 10.1182/blood-2005-11-026344 PMC1988938

[18] Åndrén-Sandberg A, Deng Y. Aspects on gallbladder cancer in 2014. Curr Opin Gastroenterol 2014; 30: 326–331. 10.1097/MOG.0000000000000068 24686434

[19] Xu WS, Parmigiani RB, Marks PA. Histone deacetylase inhibitors: molecular mechanisms of action. Oncogene 2007; 26: 5541–5552. 17694093 10.1038/sj.onc.1210620

[20] Chao OS, Goodman OB Jr. Synergistic Loss of Prostate Cancer Cell Viability by Co-inhibition of HDAC and PARP. Mol Cancer Res. 2014 8 15. [Epub ahead of print] 10.1158/1541-7786.MCR-14-0173 25127709

[21] Yamaguchi J, Sasaki M, Sato Y, Itatsu K, Harada K, Zen Y, et al Histone deacetylase inhibitor (SAHA) and repression of EZH2 synergistically inhibit proliferation of gallbladder carcinoma. Cancer Sci 2010; 101: 355–362. 10.1111/j.1349-7006.2009.01387.x 19860841 PMC11159376

[22] Bajbouj K, Mawrin C, Hartig R, Schulze-Luehrmann J, Wilisch-Neumann A, Roessner A, et al P53-dependent antiproliferative and pro-apoptotic effects of trichostatin A (TSA) in glioblastoma cells. J Neurooncol 2012; 107: 503–516. 10.1007/s11060-011-0791-2 22270849

[23] Kitamura T, Connolly K, Ruffino L, Ajiki T, Lueckgen A, DiGiovanni J, et al The therapeutic effect of histone deacetylase inhibitor PCI-24781 on gallbladder carcinoma in BK5.erbB2 mice. J Hepatol 2012; 57: 84–91. 10.1016/j.jhep.2012.01.018 22326466 PMC3378818

[24] Huang S, Houghton PJ. Targeting mTOR signaling for cancer therapy. Curr Opin Pharmacol 2003; 3: 371–377. 12901945 10.1016/s1471-4892(03)00071-7

[25] Zaytseva YY, Valentino JD, Gulhati P, Evers BM. mTOR inhibitors in cancer therapy. Cancer Lett 2012; 319: 1–7. 10.1016/j.canlet.2012.01.005 22261336

[26] Carnero A. The PKB/AKT pathway in cancer. Curr Pharm Des 2010; 16: 34–44. 20214616 10.2174/138161210789941865

[27] Hay N, Sonenberg N. Upstream and downstream of mTOR. Genes Dev 2004; 18: 1926–1945. 15314020 10.1101/gad.1212704

[28] Bjornsti MA, Houghton PJ. Lost in translation: dysregulation of cap-dependent translation and cancer. Cancer Cell 2004; 5: 519–523. 15193254 10.1016/j.ccr.2004.05.027

[29] Nishioka C, Ikezoe T, Yang J, Koeffler HP, Yokoyama A. Blockade of mTOR signaling potentiates the ability of histone deacetylase inhibitor to induce growth arrest and differentiation of acute myelogenous leukemia cells. Leukemia 2008; 22: 2159–2168. 10.1038/leu.2008.243 18784743

[30] Huang WJ, Lin CW, Lee CY, Chi LL, Chao YC, Wang HN, et al NBM-HD-3, a novel histone deacetylase inhibitor with anticancer activity through modulation of PTEN and AKT in brain cancer cells. J Ethnopharmacol 2011; 136: 156–167. 10.1016/j.jep.2011.04.034 21530633

[31] Erlich RB, Kherrouche Z, Rickwood D, Lasorsa E, Azabdaftari G, Godoy A, et al Preclinical evaluation of dual PI3K-mTOR inhibitors and histone deacetylase inhibitors in head and neck squamous cell carcinoma. Br J Cancer 2012; 106: 107–115. 10.1038/bjc.2011.495 22116303 PMC3251846

[32] Ellis L, Ku SY, Ramakrishnan S, Lasorsa E, Azabdaftari G, Godoy A, et al Combinatorial antitumor effect of HDAC and the PI3K-Akt-mTOR pathway inhibition in a Pten defecient model of prostate cancer. Oncotarget 2013; 4: 2225–2236. 24163230 10.18632/oncotarget.1314 PMC3926822

[33] Gupta M, Ansell S, Novak A, Kumar S, Kaufmann SH, Witzig TE. Inhibition of histone deacetylase overcomes rapamycin-mediated resistance in diffuse large B-cell lymphoma by inhibiting Akt signaling through mTORC2. Blood 2009; 114: 2926–2935. 10.1182/blood-2009-05-220889 19641186 PMC2756203

